# Noise Reduction Effect of Multiple-Sampling-Based Signal-Readout Circuits for Ultra-Low Noise CMOS Image Sensors

**DOI:** 10.3390/s16111867

**Published:** 2016-11-06

**Authors:** Shoji Kawahito, Min-Woong Seo

**Affiliations:** Research Institute of Electronics, Shizuoka University, Shizuoka 432-8011, Japan; mwseo@idl.rie.shizuoka.ac.jp

**Keywords:** ultra low noise, multiple correlated double sampling, correlated multiple sampling, correlated double sampling, differential averager, CMOS image sensor, readout noise, *1/f* noise, RTS noise, noise analysis

## Abstract

This paper discusses the noise reduction effect of multiple-sampling-based signal readout circuits for implementing ultra-low-noise image sensors. The correlated multiple sampling (CMS) technique has recently become an important technology for high-gain column readout circuits in low-noise CMOS image sensors (CISs). This paper reveals how the column CMS circuits, together with a pixel having a high-conversion-gain charge detector and low-noise transistor, realizes deep sub-electron read noise levels based on the analysis of noise components in the signal readout chain from a pixel to the column analog-to-digital converter (ADC). The noise measurement results of experimental CISs are compared with the noise analysis and the effect of noise reduction to the sampling number is discussed at the deep sub-electron level. Images taken with three CMS gains of two, 16, and 128 show distinct advantage of image contrast for the gain of 128 (noise(median): 0.29 e^−^_rms_) when compared with the CMS gain of two (2.4 e^−^_rms_), or 16 (1.1 e^−^_rms_).

## 1. Introduction

Since the introduction of the concept of active-pixel CMOS image sensors (CISs) using in-pixel charge transfer [1,2], CISs have been recognized as image sensors suitable for low-light level imaging, and the introduction of pinned photodiodes in four-transistor (4T) active-pixel CISs has enabled overall image quality control for low-light-level imaging, including those for low dark current, fewer white defects, and no image lag [3,4,5]. Since the read noise performance of CISs is determined by many factors which are controlled by process, device, and circuit technologies, the read noise of CISs with pinned photodiodes is gradually reduced in the past twenty years as new techniques and technologies are introduced. In the CIS with pinned photodiodes reported in 2001, the read noise was 13.5 e^−^ [6]. Several CISs with sub-electron [7,8,9] and deep sub-electron noise [10,11,12] levels have been reported recently, and the best noise level has reached below 0.3 e^−^ [13,14,15]. In an active pixel device called DEPFET with non-destructive multiple readouts of the pixel output, very low noise level of 0.25 e^−^ [16] and 0.18 e^−^ [17] have been attained. Roughly speaking, the read noise of CISs is reduced down to one-fiftieth in the past 15 years. High conversion gain is definitely the most important factor for realizing the low read noise. However, a deep sub-electron noise level is not realized without the help of readout-circuit techniques with a high noise reduction capability. For instance, a column high-gain pre-amplifier before an analog serial readout or a column analog-to-digital conversion (ADC) is an effective technique for low-noise CISs [18,19,20]. A very low noise level of 1.5 e^−^_rms_ is demonstrated in a pinned-photodiode CIS using a high-gain (gain = 32) column amplifier [18]. For further efficient noise reduction, high-gain pre-amplification using multiple sampling of the pixel output is becoming another important technique for low-noise CISs. A multiple sampling technique known as Fowler sampling is used for reading, non-destructively, the outputs of infrared light image sensors [21], and a technique called multiple correlated double sampling (MCDS) [22], or correlated multiple sampling (CMS), is used for a pixel detector for high-energy particles [22] and column readout circuits for low-noise CISs [23,24,25]. The authors have recently applied this technique to an experimental image sensor using high-conversion gain pixels and a large sampling number of 128, and deep sub-electron noise level of 0.27 e^−^_rms_ has been attained [15].

In this paper, to reveal how the column CMS circuits, together with high-conversion-gain pixels and low-noise transistors, realizes deep sub-electron read noise levels in our previous implementation [15], the read noise of signal readout chain from the pixel to column ADC is analyzed and the noise components of the pixel and column amplifiers as a function of the sampling number (=gain) are examined to clarify the dominant noise component at high gain. The noise measurement results of the experimental CIS chip are compared with the noise analysis and the noise reduction effect to the sampling number is discussed. The noise reduction effect as a function of the sampling number is also evaluated by images taken by different CMS gains, and the advantage of image quality with the deep sub-electron noise level is demonstrated.

## 2. Signal Readout Architecture for Ultra-Low-Noise CISs

### 2.1. Active Pixel Sensors for High-Conversion Gain

**Figure 1:**
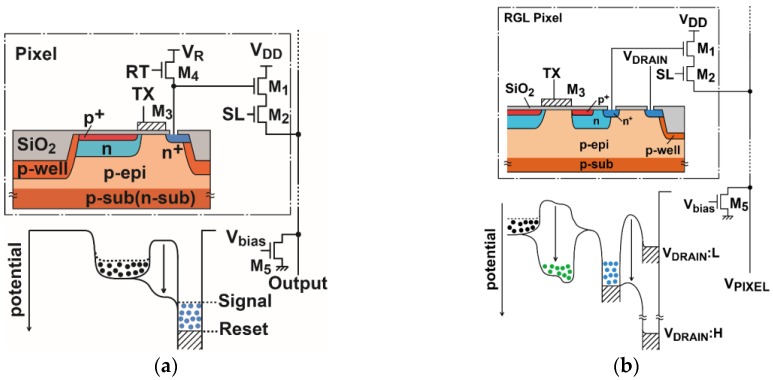
High conversion gain pixels. (**a**) 4T pixel with a pinned photodiode; and (**b**) an RGL high conversion gain pixel.

Two types of active pixel sensors (APSs), as shown in Figure 1, are used here for realizing ultra-low-noise CISs together with high-gain column readout circuits. One (Figure 1a) is the well-known APS with four transistors for a source follower (M_1_), pixel selection (M_2_), charge transfer (M_3_), and charge resetting (M_4_). The other (Figure 1b) is a special type of APS for higher conversion gain with three transistors and a reset-gateless (RGL) charge resetting technique [15,26]. Both pixels use a pinned photodiode for low dark current and signal readout with perfect charge transfer. In Figure 1a, the size of transistors, wiring, and size of floating diffusion (FD) are carefully designed to minimize the parasitic capacitance of the floating diffusion node and maximize the conversion gain. In Figure 1b, a very high conversion gain is expected because of small parasitic capacitance at the FD node not only by optimizing transistor size and wiring, but also by using a structure to reduce parasitic capacitance due to transistors. To reduce the capacitance from the gate of M_3_ to FD, a depleted potential saddle is created between the transfer gate and the FD [25]. To eliminate the capacitance of the reset transistor, the reset transistor is removed and the resetting of charge in the FD is done by pulling the drain junction to a very high level.

### 2.2. Column Readout and ADC Circuits Using Multiple Sampling

**Figure 2:**
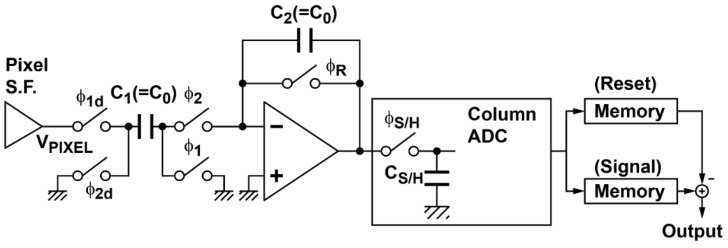
Schematic diagram of the column readout circuits using multiple sampling for low-noise readout.

**Figure 3:**
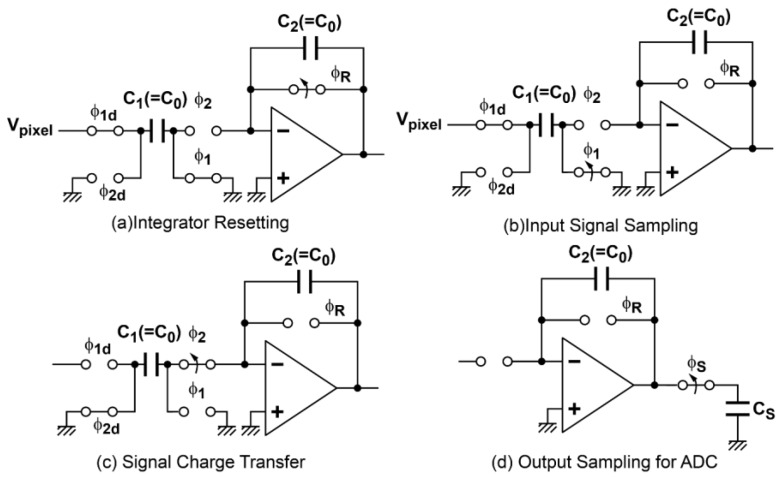
Phase diagram of the column CMS readout circuits.

**Figure 4:**
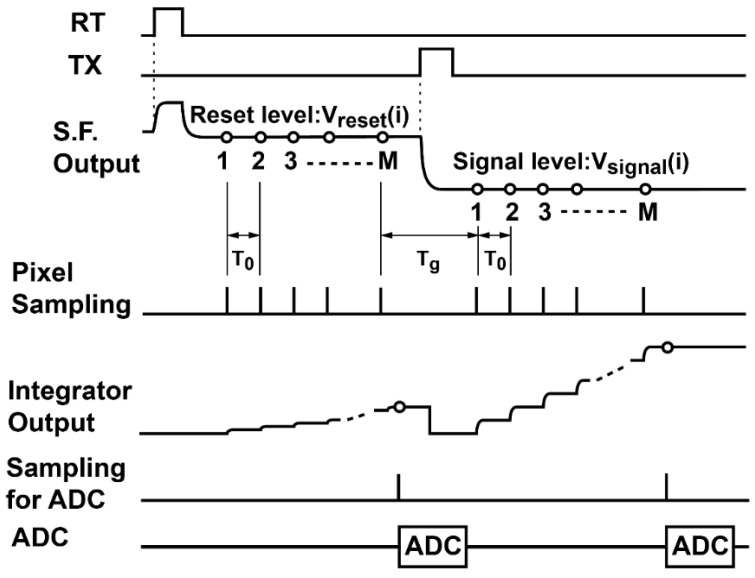
Timing diagram of the CMS.

A column readout circuit using multiple sampling is shown in Figure 2. The column correlated multiple sampling (CMS) is implemented with a switched-capacitor (SC) integrator. The operation phase diagram and timing diagram of the column CMS circuits are shown in Figure 3 and Figure 4, respectively. At the beginning, the capacitor C_2_ of the integrator is reset by turning the on switch controlled by φ_R_ as shown in Figure 3a, while the RT in the pixel in the case of the 4T pixel is set to high for resetting the FD node of the pixel. Then, for multiple sampling of the reset level, the pixel output is sampled by the capacitor C_1_ with switches controlled by φ_1_ and φ_1d_ as shown in Figure 3b and the charge in C_1_ is transferred to C_2_ as shown in Figure 3c by turning switches controlled by φ_2_ and φ_2d_ on. By repeating this operation of Figure 3b,c *M* times, the *M* samples of the reset level are integrated over in the integrator. The resulting output of the integrator after *M*-time sampling is given by $G_{I}\times M\times \;\bar{V_{reset}}$, where $\bar{V_{reset}}$ is the average of the reset level of the pixel output and $G_{I}=C_{1}/C_{2}$ is the gain of the integration in one cycle. This integrator output is sampled by a sample-and-hold capacitor and converted to an *n*-bit digital code by the *n*-bit column ADC. Similarly, after the charge transfer from the photodiode (PD) to FD by opening the charge transfer (TX) gate, the photo-signal level of the pixel output is sampled *M* times and the *M* samples are integrated over in the integrator. The resulting output after *M*-time sampling is given by $G_{I}\times M\times \bar{V_{signal}}$, where $\bar{V_{signal}}$ is the average of the photo-signal level of the pixel output. This integrator output is also sampled by a sample-and-hold capacitor and converted to an *n*-bit digital code by the *n*-bit column ADC. After the A/D conversion of the integrator output for the reset and photo-signal levels, the difference of those stored in two *n*-bit memories for reset and signal levels is taken in the digital domain to perform the correlated double sampling (CDS) for cancelling the pixel fixed pattern noise (FPN) and reset noise. This CMS processing, which is a combination of *M*-time sampling and integration in the analog domain, and the CDS in digital domain, has high suppression effects of thermal and *1/f* noise and a strong effect of cancelling vertical FPN (VFPN) of CISs, which is caused by the offset deviation of the column readout circuits. The sampling number of the readout circuits based on the CMS technique should be carefully chosen by their applications, e.g., the sensor operations can be determined by following the desired capabilities for applications: (1) high sensitivity with a relatively low frame rate; and (2) high operation speed with an allowable noise level.

## 3. Noise Analysis of Readout Circuits with Multiple Sampling

### 3.1. Modeling of Noise Sources: Pixel Source Follower and Column Amplifier

**Figure 5:**
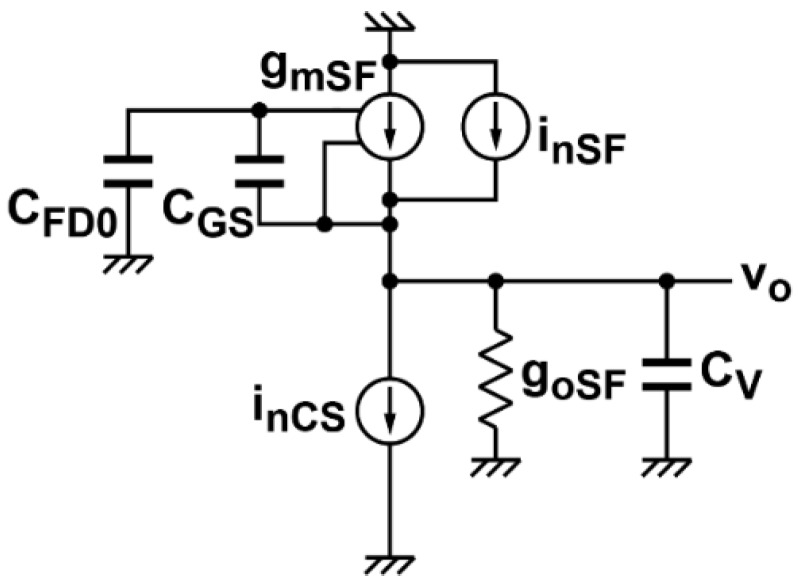
Equivalent circuit of the pixel source follower for noise analysis.

An equivalent circuit of the active pixel for the noise modeling is shown in Figure 5. The pixels with high conversion gain shown in Figure 1a,b can use the same equivalent circuit of Figure 5. The conversion gain of the pixel using a source follower amplifier, *G_cSF_*, is given by:

(1) $$G_{cSF}=\frac{qG_{SF}}{C_{FD0}+(1-G_{SF})C_{GS}}$$

where *G_SF_* is the source follower gain, *C_GS_* is the gate-to-source capacitance of the in-pixel transistor M_1_, *C_FD_*_0_ is the capacitance at the floating diffusion node other than the term due to *C_GS_* and *q* is the elementary charge. The source follower DC gain *G_SF_* is given by:

(2) $$G_{SF}=\frac{g_{mSF}}{g_{oSF}+g_{mSF}}$$

where *g_mSF_* is the transconductance of M_1_ and *g_oSF_* is the output conductance of the source follower, which includes the equivalent conductance component due to the body bias effect of M_1_ and the output conductance of M_1_ and the current-source load M_4_. The gain of the source follower is typically 0.8–0.9. The noise power (squared current) spectrum density *S_InSF_* measured at the source follower output [27], including the thermal and *1/f* (flicker) noise sources, is expressed as:

(3) $$S_{InSF}=4k_{B}T\xi_{SF}g_{mSF}+\frac{K_{fSF}}{f}\varsigma_{SF}g_{mSF}{}^{2}$$

where *k_B_* is the Boltzmann constant, *T* is the absolute temperature, *f* is the frequency. *ξ_SF_* is the excess thermal noise factor of the source follower given by:

(4) $$\xi_{SF}=\xi_{P}+\frac{g_{mCS}}{g_{mSF}}\xi_{CS}$$

where *ξ_P_* and *ξ_CS_* are the excess noise factor of M_1_ and M_4_, respectively. *ζ_SF_* is the flicker noise factor to include the influence of the current-source load given by:

(5) $$\varsigma_{SF}=1+\frac{K_{fCS}}{K_{fSF}}{\left(\frac{g_{mCS}}{g_{mSF}}\right)}^{2}$$

where *K_fSF_* and *K_fCS_* are the flicker noise coefficients of M_1_ and M_4_, respectively.

**Figure 6:**
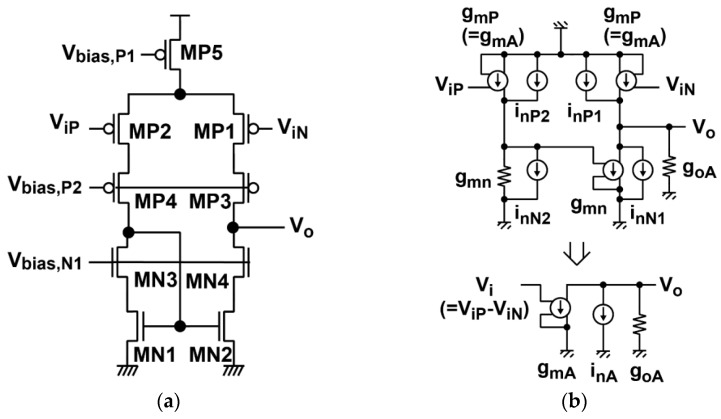
Operational amplifier used in the integrator and its equivalent circuits for noise calculation. (**a**) Circuit schematic; and (**b**) the equivalent circuit for noise analysis.

As for an operational amplifier (op-amp) used in the integrator, a high-gain single-pole op-amp using telescopic cascode or folded cascode topology can be used. Figure 6a,b show a telescopic cascode op-amp used in the column readout circuits of this CIS design and its equivalent circuit for noise analysis. In the telescopic cascode op-amp of Figure 6a, the noise of transistors MP5, MP3, MP4, MN4, and MN3 is ignored in the equivalent circuit of Figure 6b. Then the equivalent noise power spectrum *S_InA_* measured at the source follower output, including the thermal and *1/f* (flicker) noise sources, is expressed as:

(6) $$S_{InA}=4k_{B}T\xi_{A}g_{mA}+\varsigma_{A}\frac{K_{fA}}{f}g_{mA}{}^{2}$$

where *ξ*_A_ is the excess thermal noise factor of the op-amp, which includes the influence of all of the transistors given by:

(7) $$\xi_{A}=2\left(\xi_{PA}+\frac{g_{mNA}}{g_{mA}}\xi_{NA}\right)$$

where *ξ_PA_* and *ξ_CS_* are the excess noise factors of MP_1_ (MP_2_) and MN_1_ (MN_2_), respectively. *ζ*_A_ is the flicker noise factor to include the influence of all the transistors given by:

(8) $$\varsigma_{A}=2\left(1+\frac{K_{fNA}}{K_{fPA}}{\left(\frac{g_{mNA}}{g_{mA}}\right)}^{2}\right)$$

where *K_fSF_* and *K_fCS_* are the flicker noise coefficient of M_1_ and M_4_, respectively, and the *g_mA_* and *g_mNA_* are the transconductances of MP_1_ (MP_2_) and MN_1_ (MN_2_), respectively.

### 3.2. Analysis of Noise Components of Readout Circuits

**Figure 7:**
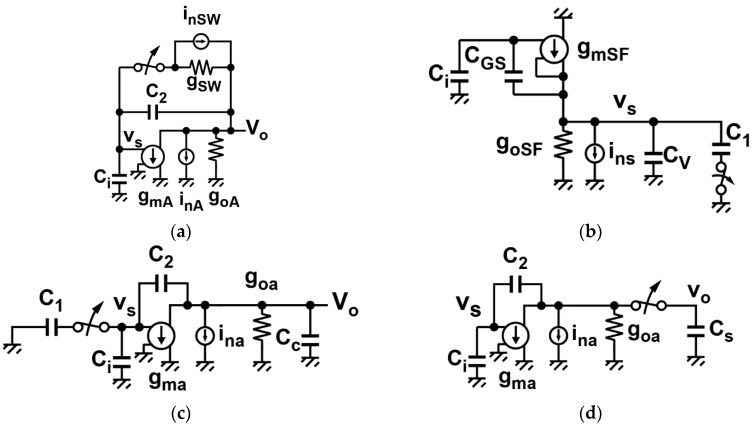
Equivalent circuits for noise calculation at four phases of Figure 3. (**a**) Integrator resetting (Figure 3a); (**b**) input signal sampling (Figure 3b); (**c**) signal charge transfer (Figure 3c); and (**d**) integrator output sampling for ADC (Figure 3d).

During the signal readout process from the pixel output sampling to A/D conversion, the readout circuits’ noise is superimposed on the photo signal at each phase of operation of the CMS readout circuits. The equivalent circuits for noise calculation at each phase of Figure 3 are shown in Figure 7.

#### 3.2.1. Reset Noise of the Integrator

During the resetting phase of the integrator, the thermal noise of the switch by φ_R_ is sampled in the capacitor *C*_2_ and appears at the integrator output. The noise due to the operational amplifier and the influence of input capacitance of the amplifier *C_i_* can be neglected in this phase. Then this noise power component denoted by *P_nT,rst_* is approximately given by:

(9) $$P_{nT,rst}=2\frac{k_{B}T}{C_{2}}$$

Due to the digital CDS operation for the output of the integrator, the resetting is done two times for the pixel reset level and signal level, and the reset noise power is increased by a factor of two, as in Equation (9).

#### 3.2.2. Thermal and *1/f* Noise in the Input Signal Sampling Phase

The equivalent circuit in the input sampling phase of the integrator is shown in Figure 7b. The major noise component in this phase is the thermal and *1/f* noise of the pixel source follower and these noises are influenced by the noise-power transfer function of the source follower. Using the equivalent circuits of Figure 5, the noise-power transfer function denoted by $|H_{nSF}(\omega ){|}^{2}$ is given by:

(10) $$|H_{nSF}(\omega ){|}^{2}=\frac{G_{nSF}{}^{2}}{1+{\left(\omega /\omega_{cSF}\right)}^{2}}$$

where *G_nSF_* is the noise gain factor of the source follower based on the fact that the noise current due to M_1_ and M_5_ (current source load) is amplified by the positive feedback effect of C_GS_ of the source follower and is expressed as [28]:

(11) $$G_{nSF}=\frac{G_{SF}(C_{FD0}+C_{GS})}{C_{FD0}+(1-G_{SF})C_{GS}}$$

and *ω_cSF_* is the cutoff angular frequency of the source follower with the load capacitance of *C_v_* and sampling capacitance of *C*_1_ which is given by:

(12) $$\omega_{cSF}=\frac{g_{mSF}}{G_{nSF}(C_{V}+C_{1})}$$

Due to the positive feedback effect caused by *C_GS_*, the actual transconductance of the source follower is reduced by the same factor of the noise gain *G_nSF_*.

In the phase diagram of the CMS readout circuits (Figure 3b), the noise of the pixel source follower is sampled in the capacitor *C*_1_, and then the sampled noise is transferred to *C*_2_. This operation is done *M* times for both reset and signal levels, and the difference of the integrator output after A/D conversion is taken for the digital CDS. As a result, the noise in this phase, which is finally contained in the digital-domain signal is calculated with the transfer functions of the CMS and the source follower. The noise components in this phase, the thermal (*P_nT,smpl_*) and the *1/f* (*P_nF, smpl_*) noises, are expressed as:

(13) $$P_{nT,smpl}+P_{nF,smpl}=\int_{-\infty }^{\infty }\frac{S_{InSF}}{g_{mSF}{}^{2}}{\left|H_{nSF}(\omega )\right|}^{2}{\left|H_{CMS}(\omega )\right|}^{2}df$$

where $|H_{CMS}(\omega ){|}^{2}$ is the power transfer function of the CMS given by [29,30]:

(14) $$|H_{CMS}(\omega ){|}^{2}=\frac{4\sin^{2}(M\omega T_{0}/2)\sin^{2}((M+M_{G}-1)\omega T_{0}/2)}{\sin^{2}(\omega T_{0}/2)}$$

For the thermal noise component of Equation (13), a sampled noise of one cycle is calculated by the noise power spectrum and transfer function of the source follower. After the CMS operation, the noise power sampled and accumulated with 2*M* times in the integrator is given by:

(15) $$P_{nT,smpl}=2{G_{I}}^{2}M{G_{nSF}}^{2}\xi_{SF}\frac{k_{B}T}{g_{mSF}}\omega_{cSF}=\frac{2{G_{I}}^{2}MG_{nSF}\xi_{SF}k_{B}T}{C_{V}+C_{1}}$$

For the *1/f* noise component, Equation (13) can be written as:

(16) $$P_{nF,smpl}={G_{I}}^{2}M^{2}{G_{nSF}}^{2}\varsigma_{SF}K_{fSF}\int_{0}^{\infty }\frac{{\left|H_{nSF}(\omega )\right|}^{2}{\left|H_{CMS}(\omega )\right|}^{2}}{G_{nSF}{}^{2}M^{2}f}df$$

The integral in Equation (16) is a noise reduction factor of the CMS to *1/f* noise and is defined by:

(17) $$F_{CMS}(M,M_{G},x_{c})=\int_{0}^{\infty }\frac{4\sin^{2}(Mx/2)\sin^{2}((M+M_{G}-1)x/2)}{M^{2}x(1+{\left(x/x_{c}\right)}^{2})\sin^{2}(x/2)}dx$$

with the definition of $x=\omega T_{0}$ and $x_{c}=\omega_{cSF}T_{0}$. Then Equation (16) can be expressed as:

(18) $$P_{nF,smpl}=G_{I}{}^{2}M^{2}G_{nSF}{}^{2}\varsigma_{SF}K_{fSF}F_{CMS}(M,M_{G},\omega_{cSF}T_{0})$$

The factor of the *1/f* noise reduction for the CMS for a large *M* becomes almost the same as that for the case of the noise reduction technique called the differential averager using continuous integration [31]. The ratio of *M_G_* to *M* is denoted by *R_G_*, i.e., $R_{G}=M_{G}/M$. Then the noise reduction factor of the CMS can be approximated by a noise reduction factor of the differential averager F_DA_, which is a function of *R_G_* only and is given by [31]:

(19) $$\frac{F_{DA}(R_{G})}{2}=\frac{1}{2}R_{G}{}^{2}\ln R_{G}+\frac{1}{2}{\left(2+R_{G}\right)}^{2}\ln(2+R_{G})-{\left(1+R_{G}\right)}^{2}\ln(1+R_{G})$$

**Figure 8:**
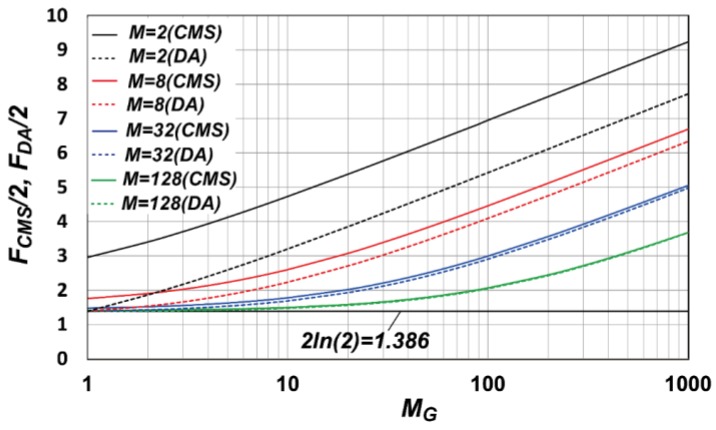
Noise reduction factor of the CMS, *F_CMS_*, and differential averager, *F_DA_*, as a function of *M_G_* and *M*.

For $R_{G}<<1$, it is approximated as $F_{DA}(R_{G})/2=2\ln(2)≅1.386$. Equation (19) is a useful equation for calculating the *1/f* noise after the CMS operation without numerical calculation of the integration, as is done in Equation (17). For a large *M*, *F_CMS_* can be exactly approximated by *F_DA_*. However, for a small *M*, *F_CMS_* become larger than *F_DA_*. Figure 8 shows the noise reduction factor of the CMS, *F_CMS_*, and the differential averager, *F_DA_*, as a function of *M_G_*, for the multiple sampling number (*M*) of two, eight, 32, and 128. *x_c_* of 30 is assumed. For efficient noise reduction of the *1/f* noise, the ratio of *M_G_* to *M* or *R_G_* must be kept as small as possible and, from Figure 8, the noise increase is less than 5% if *M_G_* is less than 10% of M. In case that *M_G_* is much larger than *M*, it must be noted that the noise reduction effect of the CMS becomes considerably worse than the ideal factor of 2ln(2)≅1.386.

#### 3.2.3. Thermal and *1/f* Noise in the Signal Charge Transfer Phase

In charge transfer phase of Figure 3c, the signal charge sampled in *C*_1_ is transferred to *C*_2_, and then *C*_1_ is disconnected from the input of the op-amp. At this instance, a noise charge caused by the noise of the op-amp used in the SC integrator is sampled in *C*_1_. The sampled noise charge in *C*_1_ is lost in the next input sampling phase. As a result, a noise charge, which is the same amount but opposite polarity as the noise charge in *C*_1_, remains in *C*_2_ of the SC integrator. This noise component is generated in every cycle of the multiple-sampled integration, and the final noise component as a result of the CMS operation is calculated with the noise power transfer function of the SC integrator and CMS using the equivalent circuit of Figure 7c. The power transfer function $|H_{nA}(\omega ){|}^{2}$ of the SC integrator to the noise source including the load and sampling capacitances is given by:

(20) $$|H_{nA}(\omega ){|}^{2}=\frac{1}{\beta_{A}{}^{2}}\frac{1}{1+{\left(\omega /\omega_{cA}\right)}^{2}}$$

where *β_A_* is the feedback factor of the SC integrator expressed as:

(21) $$\beta_{A}=\frac{C_{2}}{C_{2}+C_{1}+C_{i}}$$

and *ω_cA_* is the cutoff angular frequency of the SC integrator given by:

(22) $$\omega_{cA}=\frac{g_{mA}\beta_{A}}{C_{L,trns}}$$

where *C_L,trns_* is the load capacitance of the SC integrator in charge transfer phase given by:

(23) $$C_{L,trns}=\frac{C_{2}(C_{1}+C_{i})}{C_{2}+C_{1}+C_{i}}+C_{c}$$

In Equation (23), *C_c_* is the additional capacitance at the output for bandwidth limitation of the SC integrator. The noise components in this phase, the thermal (*P_nT,trns_*) and the *1/f* (*P_nF, trns_*) noises, are calculated by:

(24) $$P_{nT,\;trns}+P_{nF,\;trns}=\beta_{S}{}^{2}\int_{-\infty }^{\infty }\frac{S_{InA}}{g_{mA}{}^{2}}{\left|H_{nA}(\omega )\right|}^{2}{\left|H_{CMS}(\omega )\right|}^{2}df$$

where $\beta_{S}$ is the noise charge re-sampling factor when the capacitor *C*_1_ is disconnected from the charge summation node of *V_s_*, which is given by:

(25) $$\beta_{S}=\frac{C_{1}}{C_{1}+C_{2}+C_{i}}$$

The thermal noise component after the CMS operation is calculated as:

(26) $$P_{nT,trns}=2M\xi_{A}\frac{k_{B}T}{g_{mA}}\frac{\beta_{S}{}^{2}}{\beta_{A}{}^{2}}\omega_{cA}=2G_{I}{}^{2}M\xi_{A}k_{B}T\frac{\beta_{A}}{C_{L,trns}}$$

For the *1/f* noise component, Equation (24) can be written as

(27) $$P_{nF,trns}=G_{I}{}^{2}M^{2}\varsigma_{A}K_{fA}F_{CMS}(M,M_{G},\omega_{cA}T_{0})$$

using Equation (17).

#### 3.2.4. Sampled Noise of the Integrator Output for A/D Conversion

The last component is the sampled noise at the sample-and-hold circuit connected at the integrator. Equivalent circuit in this phase corresponding to the Figure 3d is shown in Figure 7d. This sample-and-hold circuit is used for column A/D conversion. If the *1/f* noise, due to the amplifier used for the ADC, is ignored because of the low-noise design of the amplifier using relatively large transistor sizes, the thermal noise component (*P_nT, ADC_*) in the A/D conversion of the integrator output is calculated by:

(28) $$P_{nT,ADC}=\int_{-\infty }^{\infty }\frac{S_{InA}}{g_{mA}{}^{2}}{\left|H_{nA2}(\omega )\right|}^{2}{\left|H_{CDS}(\omega )\right|}^{2}df$$

where $\left|H_{CDS}(\omega ){|}^{2}=4\sin^{2}(\omega T_{CDS}/2\right)$ is the power transfer function of the CDS operation and $|H_{nA}(\omega ){|}^{2}$ is the noise power transfer function of the amplifier given by:

(29) $$|H_{nA}(\omega ){|}^{2}=\frac{1}{\beta_{A1}{}^{2}}\frac{1}{1+{\left(\omega /\omega_{cA2}\right)}^{2}}$$

where *β_A_* is the feedback factor of the SC integrator in the output sampling phase expressed as:

(30) $$\beta_{A1}=\frac{C_{2}}{C_{2}+C_{i}}$$

and *ω_cA_* is the cutoff angular frequency of the SC integrator given by:

(31) $$\omega_{cA}=\frac{g_{mA}\beta_{A1}}{C_{L,ADC}}$$

The thermal noise and *1/f* noise components are calculated as:

(32) $$P_{nT,ADC}=\frac{2\xi_{A}k_{B}T}{C_{L,ADC}\beta_{A1}}$$

where *C_L,ADC_* is the load capacitance in this phase given by:

(33) $$C_{L,ADC}=\frac{C_{2}C_{i}}{C_{2}+C_{i}}+C_{s}$$

The factor of two in Equation (32) is based on the fact that the CDS operation doubles the thermal noise power. This noise component generated during the A/D conversion of the integrator output depends on the type of the A/D converter used.

#### 3.2.5. Total Noise

The total noise power referred at the output of the integrator *P_nCMS,total_*, if all of the noise components are uncorrelated from each other, is given by:

(34) $$P_{nCMS,total}=P_{n,rst}+P_{nT,smpl}+P_{nF,smpl}+P_{nT,trns}+P_{nF,trns}+P_{nT,ADC}$$

Since the gain from the charge to the integrator output is given by $G_{I}\times M\times G_{cSF}$, the input referred noise is expressed as:

(35) $$N_{nCMS,total}=\frac{\sqrt{P_{nCMS,total}}}{G_{I}MG_{cSF}}=\frac{\sqrt{P_{n,rst}+P_{nT,smpl}+P_{nF,smpl}+P_{nT,trns}+P_{nF,trns}+P_{nT,ADC}}}{G_{I}MG_{cSF}}$$

To explicitly show the contribution of the noise components as noise-equivalent charge, the total input referred noise is expressed as:

(36) $$N_{nCMS,total}=\sqrt{N_{n,rst}{}^{2}+N_{nT,smpl}{}^{2}+N_{nF,smpl}{}^{2}+N_{nT,trns}{}^{2}+N_{nF,trns}{}^{2}+N_{nT,ADC}{}^{2}}$$

where:

(37) $$N_{n,rst}=\frac{\sqrt{P_{n,rst}}}{G_{I}MG_{cSF}}=\frac{1}{G_{I}MG_{cSF}}\sqrt{\frac{2k_{B}T}{C_{2}}}$$

(38) $$N_{nT,smpl}=\frac{\sqrt{P_{nT,smpl}}}{G_{I}MG_{cSF}}=\frac{1}{\sqrt{M}G_{cSF}}\sqrt{\frac{2G_{nSF}\xi_{SF}k_{B}T}{C_{V}+C_{1}}}$$

(39) $$N_{nF,smpl}=\frac{\sqrt{P_{nF,smpl}}}{G_{I}MG_{cSF}}=\frac{G_{nSF}}{G_{cSF}}\sqrt{2\varsigma_{SF}K_{fSF}F_{CMS}}$$

(40) $$N_{nT,trns}=\frac{\sqrt{P_{nT,trns}}}{G_{I}MG_{cSF}}=\frac{1}{\sqrt{M}G_{cSF}}\sqrt{\frac{2\xi_{A}k_{B}T\beta_{A}}{C_{L,trns}}}$$

(41) $$N_{nF,trns}=\frac{\sqrt{P_{nF,trns}}}{G_{I}MG_{cSF}}=\frac{1}{G_{cSF}}\sqrt{2\varsigma_{A}K_{fA}F_{CMS}}$$

and

(42) $$N_{nT,ADC}=\frac{\sqrt{P_{nT,ADC}}}{G_{I}MG_{cSF}}=\frac{1}{G_{I}MG_{cSF}}\sqrt{\frac{2\xi_{A}k_{B}T}{\beta_{A1}C_{L,ADC}}}$$

There are three-types of noise components in the CIS with the CMS readout circuits. The first type is the component whose noise amplitude is reduced by a factor of *M*, as in Equations (37) and (42). These noise components are effectively reduced by increasing the gain *M* and the total noise is almost unaffected for a large gain. The second type is the component whose noise amplitude is reduced by a factor of $\sqrt{M}$, as in Equations (38) and (40), and dominates the total noise for the middle-gain region. The third type are the components which have a weak dependency on *M*, as in Equations (39) and (41).

### 3.3. Noise Calculation for the Designed Ultra-Low-Noise CIS

**Figure 9:**
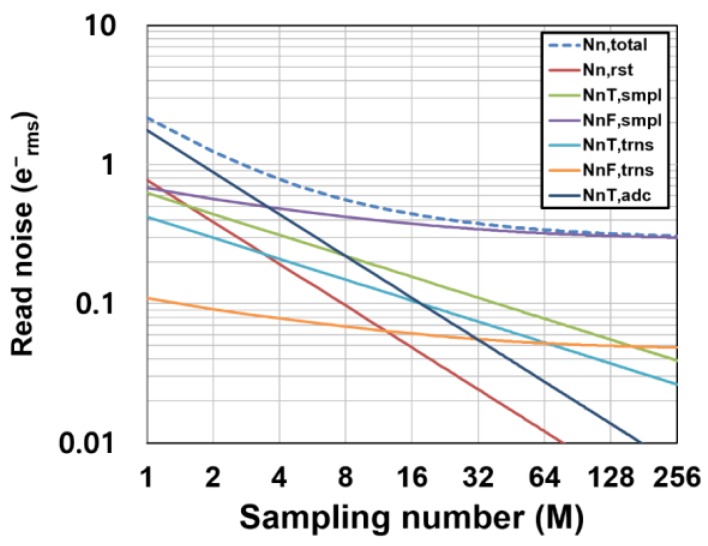
Noise components as a function of the sampling number in the CMS.

As described in Section 4, an experimental CIS chip with ultra-low-noise performance is designed and implemented. Using the device parameters used for the design of the CIS chip, the noise components of the readout circuits and the resulting total noise are calculated. Figure 9 shows an example of noise calculation of the CIS using the RGL pixels. The parameters used in this noise calculation are given in Table 1. Table 1 contains parameters for the RGL pixel and the conventional 4T pixel shown in Figure 1. The capacitances are those used for the design of the CIS chip, and the excess noise factors are calculated with the well-known characteristics of the excess noise factor as a function of channel length of nMOS transistor [32]. The *1/f* noise parameters for the amplifier design are calculated with the measured data supplied as the process design kit (PDK) from the CIS foundry. Since no measurement data on the small-size in-pixel transistors are supplied by the PDK, it is estimated by the *1/f* noise measurement data of the 3.3 V medium threshold voltage (V_T_) nMOS devices with a size of 10 μm(W)/0.55 μm(L), and the theoretical model of the *1/f* noise parameter (K*_f_*) of the nMOS transistors given by $K_{f}=k_{f}/C_{ox}{}^{2}WL$, where *k_f_* is a constant which is independent of the dimension of devices, i.e., *K_f_* is inversely proportional to the channel area (W × L). The *1/f* noise also depends on the gate bias condition, and the flicker noise coefficient is increased as the gate bias increases. With the measurement results of the *1/f* noise of the medium V_T_ device (*K_f_* = 1.4 × 10^−11^ [V^2^] @ Id = 17 μA) and the size dependency of the *1/f* noise, the flicker noise coefficients of the source follower transistors in the RGL pixel and 4T pixel are estimated as 1.0 × 10^−9^ [V^2^] and *K_f_* = 1.8 × 10^−10^ [V^2^], respectively. In this case, the source follower sizes (W/L) for the RGL and 4T pixels are 0.345 μm/0.325 μm and 0.9 μm/0.7 μm, respectively. Sometimes the optimized transistor size can lead to an increased probability that large noise, such as a random telegraph signal (RTS) noise, occurs, but a high conversion gain with the optimized SF size is more beneficial to achieve the low-noise performance. Extremely large noise generated by a smaller transistor size can be overcome by the advanced process technologies and the low-noise transistors [9].

**Figure 10:**
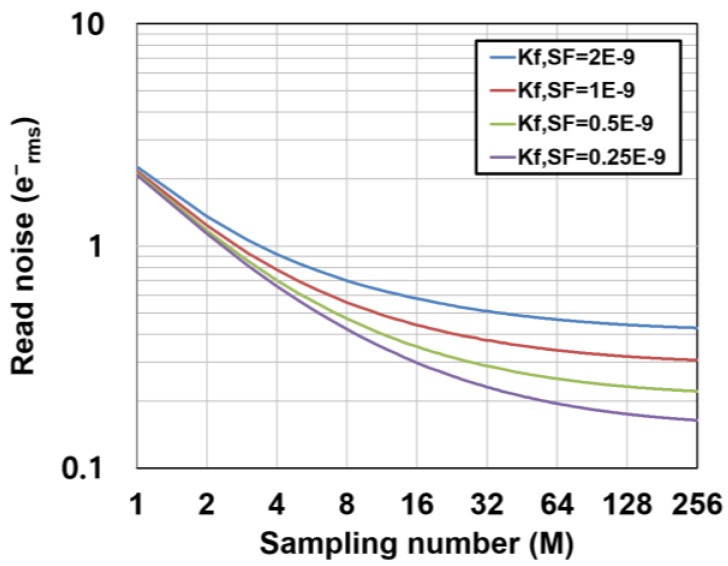
Calculated total read noise as a function of *M* and for different values of *K_f, SF_*.

Very high conversion gains of 220 μV/e^−^ and 135 μV/e^−^ are assumed for the RGL and 4T pixels, respectively, in order to compare with the experimental results described in Section 4. A *M_G_* of 16 is assumed. As shown in Figure 9, for the low-gain region (*M*: 1–4), the read noise is determined by the ADC noise. This component rapidly decreases by increasing *M* as a function of 1/*M*. In the medium-gain region (*M*: 4–16), the noise is dominated by the mixture of noise components including the thermal noise components. For the high-gain region (*M*: larger than 32), the read noise is dominated by the *1/f* noise of the pixel source follower, and because the *1/f* noise component has a slight dependency on *M* for a large *M*, the read noise approaches to the lowest limit of noise reduction. The achievable noise level for a large *M* depends on the *1/f* noise performance of the pixel source follower which is determined by the fabrication process technology and the conversion gain. A deep sub-electron noise level can be realized if a pixel with low *1/f* noise devices and high conversion gain is available. Figure 10 shows the calculated total read noise as a function of *M* and for different *1/f* noise parameters of the pixel source follower. If the target noise level is 0.2 e^−^_rms_, a very high CMS gain (*M* > 64) and a low *1/f* noise transistor (*K_f_* < 0.25 × 10^−9^ [V^2^]) is necessary if the conversion gain is unchanged for maintaining the signal dynamic range.

## 4. Implementation and Results

### 4.1. Implementation

**Figure 11:**
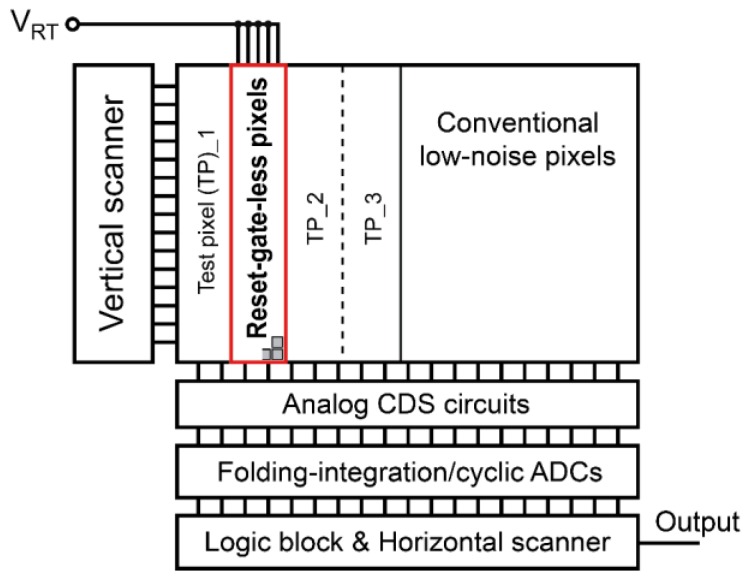
Block diagram of the experimental CIS chip.

An experimental CMOS image sensor with 32 (V) × 512 (H) RGL active pixels (Figure 1b) and 110 (V) × 512 (H) 4T active pixels (Figure 1a) has been implemented using Dongbu HiTek (Eumseong, Korea) 0.11 μm CIS technology. The block diagram of the CIS chip is shown in Figure 11. In this experimental chip, the CMS circuit is implemented as a column ADC, called the folding-integration ADC [24,25]. This ADC works as a resettable first-order delta-sigma modulator, which is based on the multiple-sampling based integrator shown in Figure 2, but has a negative feedback loop with a one-bit sub-ADC and one-bit DAC for an extended dynamic range. For instance, the output of the conventional multiple-sampling based integrator increases linearly in small input signal region, and then saturates. In the folding integration, however, the analog signal amplitude is kept to a limited range by the folding operation, while applying a high analog gain by the integration. After the folding-integration operation, the integrator output is digitized with another high-resolution ADC, called a cyclic ADC, which is implemented with the same analog circuits as the folding-integration ADC. This column ADC using multiple sampling and the digital CDS has almost the same noise reduction effect as the CMS circuits described in Section 2.

**Figure 12:**
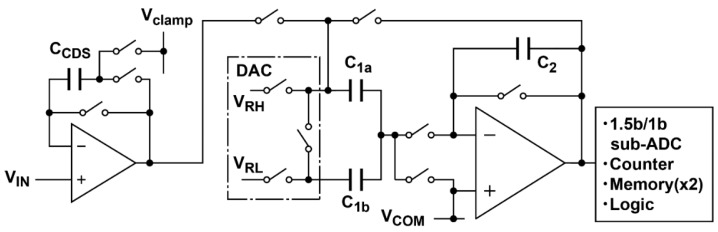
Column analog CDS and ADC circuits.

The noise analysis given in Section 3 is based on a simplified and more general type of the CMS readout circuits. This simplified analysis is useful for understanding the contribution of noise components at different gain settings of the CMS. To compare the noise measurement results and the noise calculated for the readout circuits actually implemented a few modifications to the noise model are necessary. In the actual implementation, an analog CDS circuit is used in front of the column ADC, as shown in Figure 12. This is for clamping the pedestal level (or reset level) to a fixed voltage level, which is close to the bottom reference level of the ADC to maximize the available voltage range. The reset noise is generated in the analog CDS circuits, but it is cancelled by the final digital CDS operation in the digital domain [33]. The CMS circuits actually used are implemented as a folding integration ADC, of which the analog core is also used for the cascaded A/D conversion using the cyclic ADC, as shown in Figure 12. To include the noise due to the analog CDS circuit, and the influence of the noise increase due to another sampling capacitor *C*_1*b*_, the thermal noises in the input sampling phase and charge transfer phase given by Equations (15) and (26), respectively, are modified as:

(43) $$P_{nT,smpl2}=2G_{I}{}^{2}Mk_{B}T\left(\frac{G_{nSF}\xi_{SF}}{C_{V}}+\frac{\xi_{CA}+1}{C_{1}}\right)$$

where *ξ_CA_* is the excess thermal noise factor of the op-amp for the analog CDS amplifier, and:

(44) $$P_{nT,trns2}=2M\xi_{A}\frac{k_{B}T}{g_{mA}}\frac{\beta_{S2}{}^{2}}{\beta_{A2}{}^{2}}\omega_{cA}=2M\xi_{A}k_{B}T\frac{\beta_{S2}{}^{2}}{\beta_{A2}{}^{2}}\frac{\beta_{A2}}{C_{L,trns2}}$$

where $\beta_{S2}$ is the noise charge re-sampling factor given by:

(45) $$\beta_{S2}=\frac{2C_{1}}{2C_{1}+C_{2}+C_{i}}$$

$\beta_{A2}$ is the feedback factor given by:

(46) $$\beta_{A2}=\frac{C_{2}}{2C_{1}+C_{2}+C_{i}}$$

and *C_L,trns_*_2_ is the load capacitance:

(47) $$C_{L,trns2}=\frac{(2C_{1}+C_{i})C_{2}}{2C_{1}+C_{2}+C_{i}}+C_{c}$$

of the actually implemented CMS circuits as the floding-integration ADC using *C*_1*a*_ and *C_b_*_1_ whose capacitances are *C*_1_. The input-referred noises of these components are modified from Equations (38) and (40) as:

(48) $$N_{nT,smpl2}=\frac{\sqrt{P_{nT,smpl2}}}{G_{I}MG_{cSF}}=\frac{\sqrt{2k_{B}T}}{\sqrt{M}G_{cSF}}\sqrt{\frac{G_{nSF}\xi_{SF}}{C_{V}}+\frac{\xi_{CA}+1}{C_{1}}}$$

and

(49) $$N_{nT,trns2}=\frac{\sqrt{P_{nT,trns}}}{G_{I}MG_{cSF}}=\frac{2}{\sqrt{M}G_{cSF}}\sqrt{\frac{2\xi_{A}k_{B}T\beta_{A2}}{C_{L,trns2}}}$$

respectively.

### 4.2. Noise Reduction Effect of the CMS

**Figure 13:**
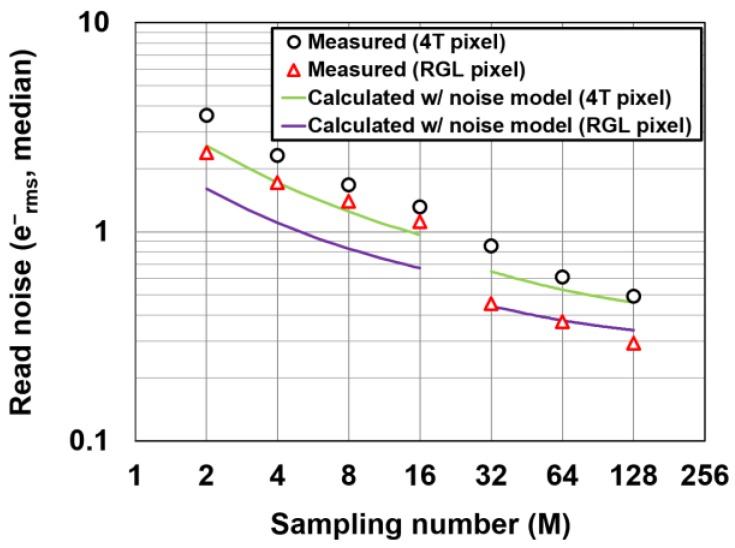
Measured noise as a function of the sampling number and the comparison with the noise calculated with the noise model.

**Figure 14:**
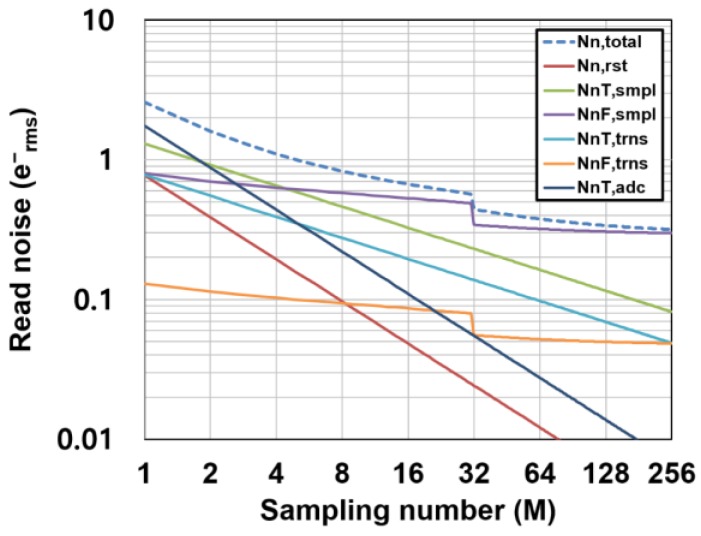
Noise components as a function of the sampling number in the folding-integration ADC.

The noise reduction effect of the CMS is experimentally demonstrated in the deep sub-electron noise region. Figure 13 shows the measured and calculated input-referred noise (noise equivalent charge) as a function of the multiple-sampling gain(the sampling number) of the CMS. The noise calculated with the noise model of the CMS circuits is also shown. The timing diagram for reading one horizontal line of the image signal and the value of *M*, *M_G_*, and the actual readout time of one horizontal line used in this measurement is shown in Figure 14 and Table 2, respectively. In order to reduce the influence of dark current, and to evaluate the noise of readout circuits only, the following data including those of Figure 13 were measured at −10 °C. Even if the read noise is measured at room temperature, the result is almost the same as the current noise level, but the total noise distribution at room temperature is slightly spread by the influence of dark current, particularly from the FD node.

**Figure 15:**
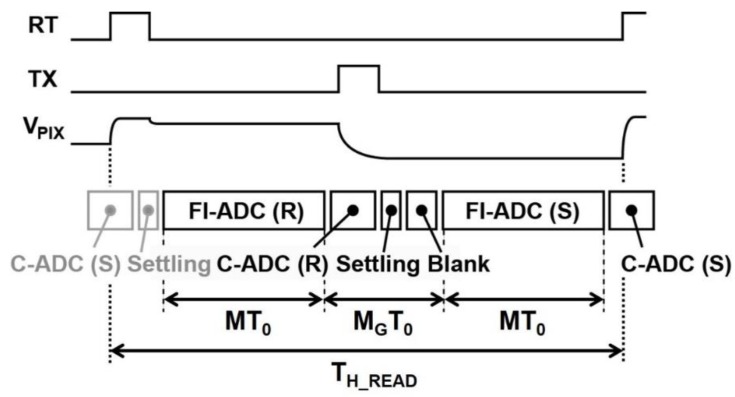
Timing diagram of signal readouts and A/D conversion.

As shown in Figure 15 and Table 2, *M_G_* for low gain (*M* = 2, 4, 8, and 16) is set to large values of more than 200. This causes a lesser *1/f* noise reduction effect, as explained in Equation (17). For high gain (*M* = 32, 64, and 128), *M_G_* of 16 is used, and a high *1/f* noise reduction effect is expected. The CMS effectively reduces the noise (median) from 3.7 e^−^ to 0.5 e^−^ for the 4T pixel, and 2.3 e^−^ to 0.29 e^−^ for the RGL pixel, respectively, by increasing the gain from two to 128. The noise calculated with the proposed model does not perfectly explain the experimental results, particularly at the low CMS gain. Since the *1/f* noise suppression capability of the CMS can be degraded by increasing the time from reset to signal samples, and the noise of the small-size transistors in the pixels does not always take the exact *1/f* noise spectrum. These can make the difference between the simulation and measurement. Another possible reason is that the noise of the cyclic ADC is not exactly modeled and other noise components, such as the noise from power supply lines of the substrate, are not included in the noise model. Such noises from power lines of the substrate are often generated due to on-chip digital switching or clocking circuits. Since these noises are not uniform in time, the irregular dependency of the noise reduction to the sampling number of the CMS, or the difference of the calculation and measurement results is likely explained. The measurement results show that the read noise can be further reduced by increasing the CMS gain. This larger dependency of the noise reduction to the CMS gain at high gain (*M* = 32, 64, and 128) when compared to the theoretical estimation is not clear, but is possibly due to the influence of the additional thermal noise components, which are not modeled in the theory, or RTS (random telegraph signal)-like noise of the in-pixel source follower. The RTS noise or RTS-like noise has a Lorentzian spectrum, or a mixture of Lorentzian spectra and the noise with such a spectrum can be reduced by band-width reduction using a higher CMS gain. The noise of the majority of pixels may take the spectrum of RTS-like noise, not that of the *1/f* noise.

**Figure 16:**
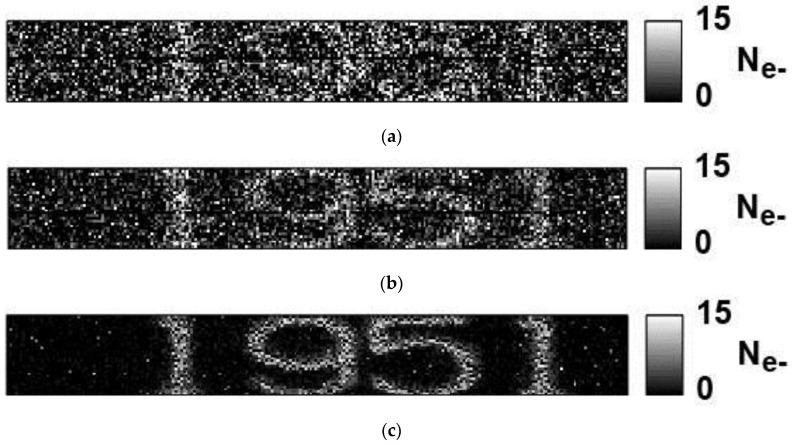
Low-light-level images with three different CMS gains (*M* = 2, 16, and 128). (**a**) *M* = 2, noise (median): 2.4 e^−^_rms_; (**b**) *M* = 16, noise (median): 1.1 e^−^_rms_; and (**c**) *M* = 128, noise (median): 0.29 e^−^_rms_.

In order to demonstrate the noise reduction effect of CMS in the deep sub-electron region, sample images are taken by three different CMS gains of two, 16, and 128, as shown in Figure 16. With these three gains of two, 16, and 128, the noise levels (median) of 2.4 e^−^_rms_, 1.1 e^−^_rms_, and 0.29 e^−^_rms_, respectively, have been obtained. The character code of “1951” in a part of the USAF (United State Air Force) test chart is used for this imaging test of three different low-noise levels and small signal photoelectron number of less than ten. When compared to the image with the noise level of 1.1 e^−^_rms_, which is the best noise level of commercially available very-low-noise CISs, the image with the noise level of 0.29 e^−^_rms_ has advantages in image contrast and recognizability of the character code. In the image with the noise level of 2.4 e^−^_rms_, it is hard to recognize the character code without prior knowledge that the character code is “1951”.

**Figure 17:**
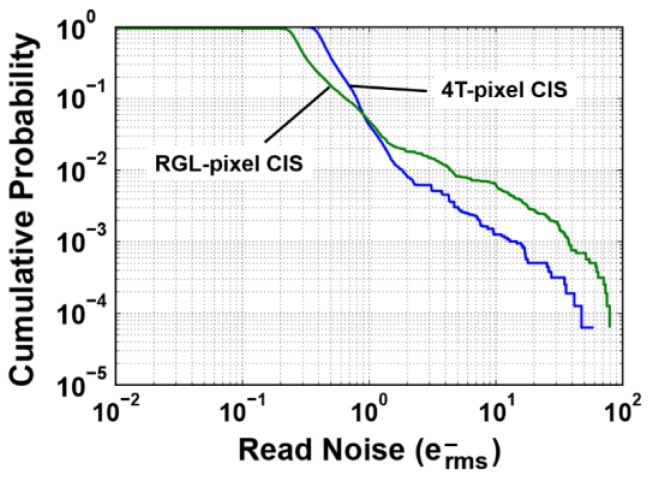
Cumulative probability plots of noise in the RGL-pixel CIS and 4T-pixel CIS.

In Figure 17, the cumulative probability plot of noise for the RGL-pixel CIS and 4T-pixel CIS is shown. The CMS gain (*M*) of 128 is used. The transistor size of the in-pixel source follower of the RGL pixel is 0.325 mm × 0.345 mm, and that of the 4T pixel is 0.7 μm × 0.9 μm. Due to the small gate area of the in-pixel source follower transistor of the RGL pixel, the population of noisy pixels with greater than 1 e^−^ is higher than that of the 4T-pixel CIS [34].

## 5. Conclusions

This paper describes a noise model for explaining the ultra-low noise level of CMOS image sensors, and the noise reduction effect of the multiple-sampling-based readout circuits used. The use of very high multiple-sampling gain of correlated multiple sampling (CMS) circuits for signal readout sufficiently reduces the noise components of readout circuits, other than the *1/f* noise of the in-pixel source follower, and the resulting noise level of CMOS image sensors can be smaller than 0.3 e^−^ using a high conversion gain pixel, high CMS gain (> 100), and a low-noise in-pixel transistor. Though the noise model does not perfectly explain the noise reduction effect of the CMS circuits, it can be used for theoretically predicting the deep sub-electron noise level in the design of CMOS image sensors by knowing the circuit and device parameters. A comparison of images taken with read noise levels of 1.1 e^−^ and 0.29 e^−^ have shown distinct merit in image contrast by reducing the read noise of the deep sub-electron noise level.

## Figures and Tables

**Table 1:** Device and circuit parameters used for noise calculations.

Parameters	Values (Conventional 4T)	Values (RGL pixel)
Temperature (K)	263	263
G_cSF_ (μV/e^−^)	135	220
G_nSF_	2.22	1.21
G_I_	0.5	0.5
C_1_ (F)	0.5 × 10^−12^	0.5 × 10^−12^
C_2_ (F)	1.0 × 10^−12^	1.0 × 10^−12^
C_V_ (F)	0.84 × 10^−12^	0.84 × 10^−12^
C_i_ (F)	0.15 × 10^−12^	0.15 × 10^−12^
C_S_ (F)	0.5 × 10^−12^	0.5 × 10^−12^
C_C_ (F)	0.5 × 10^−12^	0.5 × 10^−12^
*K_fSF_* (V^2^)	1.8 × 10^−10^	1.0 × 10^−9^
*K_fA_* (V^2^)	0.98 × 10^−11^	0.98 × 10^−11^
ξ_SF_	2.15	2.87
ξ_A_	2.25	2.25
ζ_SF_	1.01	1.01
ζ_A_	3.94	3.94

**Table 2:** *M*, *M_G_* and *T_H-READ_* used in the measurements.

M	M_G_	V_H_READ_ (μs)
2	268	172
4	264	172
8	256	172
16	240	172
32	16	57.6
64	16	96
128	16	172

## Abbreviations

The following abbreviations are used in this manuscript:

CIS	CMOS image sensor
CDS	correlated double sampling
CMS	correlated double sampling,
ADC	analog to digital converter
MCDS	multiple correlated double sampling
